# Magnetic resonance imaging at 7.0 T for evaluation of early lesions of epiphyseal plate and epiphyseal end in a rat model of Kashin-Beck disease

**DOI:** 10.1186/s12891-020-03559-w

**Published:** 2020-08-12

**Authors:** Yong Li, Pengde Kang, Zongke Zhou, Fuxing Pei, Qing He, Dike Ruan

**Affiliations:** 1 Department of Orthopedic Surgery, Sixth Medical Center of Chinese PLA General Hospital, No.6 Fucheng Road, Beijing, China; 2 Department of Orthopedic Surgery, West China Hospital, Sichuan University, No. 37, Guoxuexiang, Chengdu, China

**Keywords:** Kashin-Beck disease, Knee joint, Magnetic resonance imaging, Epiphyseal plate, Rat

## Abstract

**Background:**

Kashin–Beck disease (KBD) is a disabling osteoarticular disease involving growth and joint cartilage. Early diagnosis can effectively prevent the progress of the disease. However, the early diagnosis of it is still very difficult. Our aim was to study the knee joint lesions of a rat KBD model using ultra-high field magnetic resonance imaging (MRI) and compare it with X-ray imaging to analyze the possible MRI manifestations of KBD, and to further explore ways to determine the pathological damage of KBD in the early stage.

**Methods:**

A total of 96 Wistar rats were selected and randomly divided into 4 groups: normal diet (Group A), KBD-affected diet (Group B), normal diet+T-2 toxin (Group C), and KBD-affected diet+T-2 toxin (Group D). T-2 toxin was administered at a dose of 0.1 mg/kg/day. In the 4th week, 8th week, and 12th week, eight rats randomly selected in each group were sacrificed by cervical dislocation after undergoing X-ray and 7.0 T MRI imaging, and then knee joints were harvested, sliced, and subjected to hematoxylin-eosin (H&E) staining.

**Results:**

Characteristic image changes including of continuity interruption and early closure and fusion of epiphyseal plates were observed on T1WI in rat model of KBD. The total necrosis rates in the H&E stain of group A to group D were 4.35, 52.38, 33.3, and 73.68%, respectively. The positive rate of image change under 7.0 T MRI was 0.833 VS. that under X-ray was 0.33 (*P* = 0.001).

**Conclusions:**

MRI at 7.0 T is highly sensitive to the early pathological changes of the epiphysis, epiphyseal plate, and metaphyseal end, which can improve imaging positive rate of KBD and decrease the rate of missed diagnosis. This imaging modality can be used for research on early joint lesions and for early diagnosis of KBD.

## Background

Kashin–Beck disease (KBD) was first described by N.I. Kashin in 1861 with a detailed description provided by E.B. and A.N. Beck between 1899 and 1902 [1, 2]. It is characterized by chondrocytic necrosis, apoptosis, and cartilage and matrix degradation [3]. More than one million people [4], mostly adults, are affected by KBD in a limited endemic area that spans southeastern Siberia, crosses northern China, and ends in central Tibet.

KBD mainly invades the developing bone during endochondral ossification phase in children [5]. It first manifests as necrosis in the deep chondrocytes of epiphyseal plate cartilage [6, 7] and articular cartilage necrosis, leading to cartilage development disorder and deformity arthropathy [8–11]. As histological diagnosis is particularly difficult, clinical and imaging examinations have provided the best means of identifying KBD to date [12].

At present, the diagnosis of KBD mainly depends on the X-ray examination [13–16] which could not reflect early damage of growth plate and cartilage sensitively [17]. A latest systematic review found that the X-ray detective rate of KBD was 11% depending on the study [18]. Further research is required to identify the effective strategies for preventing and treating KBD. Magnetic resonance imaging (MRI) is an reliable approach for studying this form of osteoarthrosis due to its ability to distinguish cartilaginous tissue from bony structures [19, 20]. In particular, the alternations of normal cartilaginous epiphyses, physes, and metaphyseal marrow during growth have been evaluated extensively with MRI because precise imaging of these tissues with plain film radiographs and computed tomography scans can be difficult [21]. Evaluating metaphyseal and epiphyseal region of knee joint of rats could be really challenging because of the anatomical small size, and ultra-high field MRI may be the most reliable and useful imaging modality for evaluation of the epiphyseal plate [22]. MRI is generally accepted as the best method to show epiphyseal plate and cartilage, but there is no report about observations of early lesions of the epiphyseal plate or epiphyseal end in a rat model of KBD with 7.0 T MRI. This model of KBD induced by a low-nutrition diet and T-2 toxin exposure could demonstrate radiographic and histopathological abnormalities of the proximal epiphyseal plate and the tibial metaphysis that are very similar to the bony changes found in patients with KBD [23]. Four weeks old Wistar rats are usually selected in this model given the fact that KBD mainly invades the developing bone during endochondral ossification in children. Therefore, in this study, ultra-high field MRI technology was used to study the knee joint MRI of a rat KBD model [23] and compare with X-ray imaging to summarize and analyze the possible MRI manifestations of KBD rat model. Tthe study also aimed to explore ways to determine the pathological damage of KBD rat model in the early stage to further provide a method for etiological research on and the prevention and treatment of KBD.

## Methods

### Instruments and reagents

T-2 toxin (Trilogy, USA), Olympus microscope (Olympus, Japan), Leica RM2125 slicer (Leica Microsystems, Germany), Nikon DXM1200 camera system (Nikon, Japan), paraffin slicer (Leica, Germany), X-ray machine (Shimadzu, Japan), 7.0 T BioSpec MR imaging system (Bruker, Germany).

### Grouping of experimental animals

Ninety-six weanling Wistar rats (50% male and 50% female, 4 weeks of age and with a weight of 60–80 g) were obtained from the Center of Laboratory Animals of West China Hospital, Sichuan University, Chengdu, Sichuan, China. Rats were maintained at a temperature of 22 °C with a relative humidity of 40–70% and a 12 h light/dark cycle. Five rats were housed per box. Prior to initiation of dosing, all rats were quarantined for 1 week and evaluated for weight gain and any gross signs of disease or injury. After quarantine, the 96 rats were divided into male and female groups with 48 rats in each group. After that, based on their weight differences, they were divided into 6 blocks, and each block size was 8. Finally, the rats were randomly divided into 4 groups, 24 rats in each group by a computerized blocked randomization method. The sample size was calculated according to the statistic references and previous studies [20, 23–25]. Group A served as a control and received standard commercial feed without additives. Group B received KBD-affected feed. Group C received standard commercial feed with T-2 toxin (0.1 mg/kg/day) by intragastric administration over 5 days a week according to the KBD model study [23]. Group D received the KBD-affected feed with T-2 toxin (0.1 mg/kg/d) over 5 days a week by intragastric administration. All rats were fed tap water throughout the experimental period. The use of animals in this study was in accordance with the National Institutes of Health publication Guide for Care and Use of Laboratory Animals (NRC, 1996).

### Feed preparation

Commercial feed was prepared in accordance with the national standard of compound feed for experimental animals, mice and rats (GB14924.3–2001); KBD-affected feed was mainly prepared from highland barley, which was collected from a KBD-affected family located in Rantang County, Sichuan, P.R. China. One hundred kilograms of KBD-affected feed contained 87 kg barley, 10 kg hay powder, 1 kg salt, and 2 kg yeast.

Rats were observed twice daily for mortality and morbidity. Once prior to treatment and the other a week later, and they were individually handled and carefully examined for abnormal behavior and/or appearance regularly (dull, sparse and easily shed hair, reduced activity, and poor nutritional status). Individual body weight data were obtained twice prior to test material administration and weekly during the treatment periods, and weights were obtained at necropsy. At the 4th, 8th, and 12th weeks, eight rats in each group were sacrificed by cervical dislocation and the knee samples composed of the tibial growth cartilage and adjoining metaphyseal bone were harvested and immersed in a 4% aqueous solution of paraformaldehyde.

### Histological observation

The epiphyseal plate tissue of the rat tibias in each group was sectioned to 4-μm thickness and stained with hematoxylin and eosin (HE). Pathological changes were observed under an optical microscope. The main target of observation was the cell layer of the epiphyseal plate. The histological anatomy assessments of epiphyseal plate necrosis were performed according to the standards of the necrosis categories previously described [4]. The necroses were categorized as focus necrosis (FN), lamellar necrosis (LN), penetration necrosis (PN) and zonal necrosis (ZN). Five measurements in the epiphyseal plate on the tibial side randomly are selected for each specimen without tissue separation, and the average value is calculated. Semi-automatic image analysis method is used for measurement to analyze the tissue morphology and calculate the full thickness of epiphyseal plate (um).

### X-ray examination

After anesthesia, the rats were placed in the supine position, and an anteroposterior X-ray of the knee joint of the rats was performed by a GE 2000D mammography device. The exposure conditions were 26 kV and 18 mAS. The X-ray films were examined by two senior physicians in the imaging center, and the results were reported independently. Inconsistent results between the two physicians were resolved via consensus.

### 7.0 T MRI scanning of the rat knee joint

Axial, coronal and sagittal images of the knee joints of rats were scanned by T2WI, and the coronal positions of the knee joints of rats were further scanned by T1WI. T1WI scanning adopted an MSME sequence, and the parameters were as follows: repetition time (TR): 561 ms; echo time (TE): 14 ms; and number of excitations: 4. The Turbo RARE sequence was used for T2WI scanning. The TR was 2500 ms, the TE was 33 ms, and the number of excitations was 4. For both imaging modalities, the scanning field of view (FOV) was 4 mm × 4 mm, the matrix was 256 × 256, the layer thickness was 1 mm, and the layer spacing was 0 mm.

### Statistical methods

Statistical analyses were performed using SPSS Version 18.0 software (SPSS Inc., Chicago, IL, USA). The numeric variables were compared by using Student’s t test or One-way Analysis of Variance (ANOVA) followed by post-hoc test (Dunnett’s test) for multiple comparisons. Chi-squared test was used to compare different types of necrosis in epiphyseal plate of knee joint, and it was also used to compare the positive rates between X-ray and MRI in the experimental group at the 4th week. If the expected value in any category is less than 5, Fisher’s exact test was used. All results were considered statistically significant at *P* < 0.05.

## Results

### Feed component analysis

Test results of the main components of the feed for the experimental rats are shown in the supplementary material.

### General observations from the rats

Compared with group A, the rats in groups B-D had dull, sparse and easily shed hair, reduced activity, and poor nutritional status. No obvious deformities of the knee joints were found at any point. In the treatment groups, the articular cartilage was gray-colored, the knee joint was found a slight swollen, and the joint capsule and synovium were hyperplastic and edematous. In groups A ~ D, 1, 3, 3, and 5 rats died, respectively.

### Imaging results

#### X-ray results for the rat knee joints

**Fig. 1:**
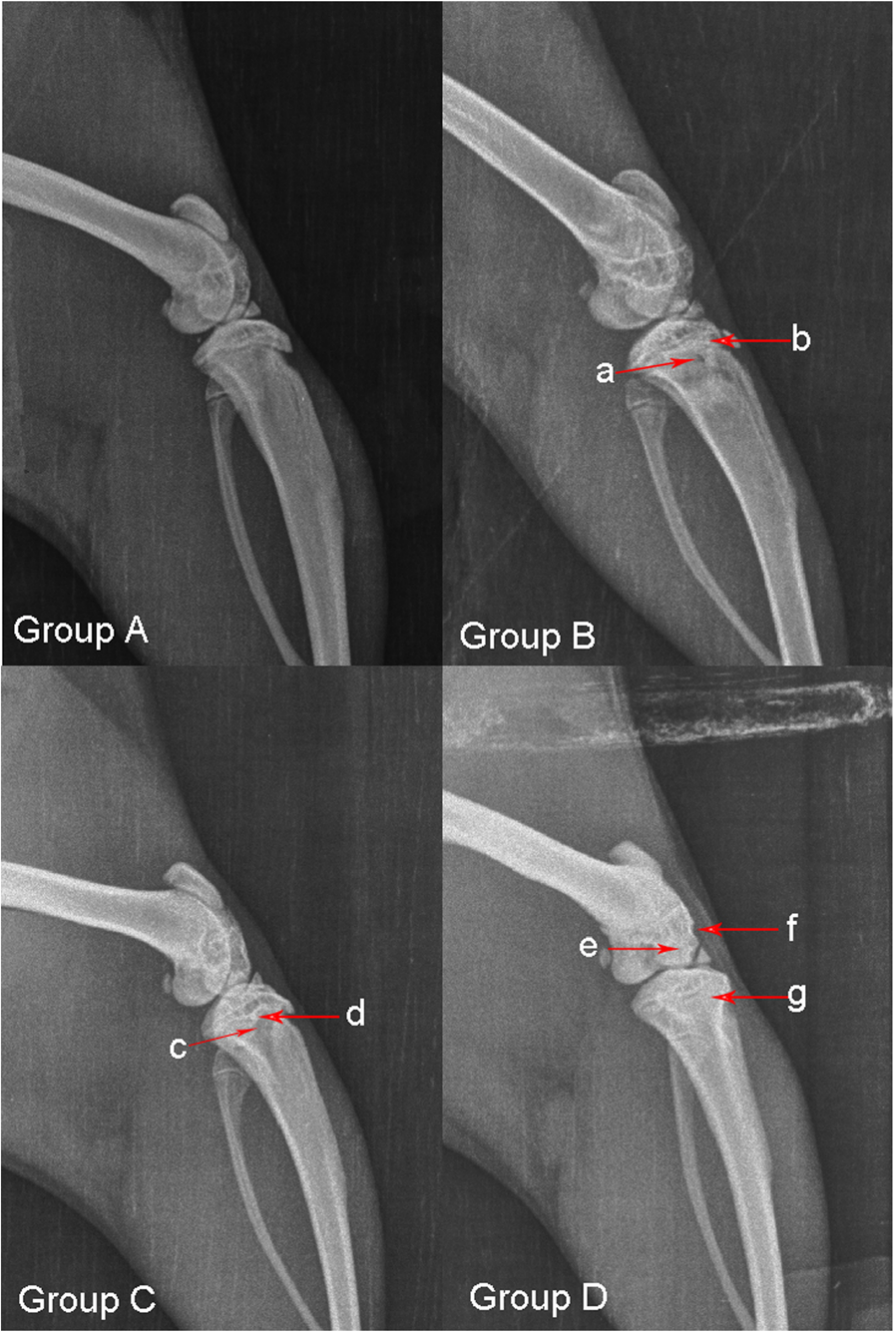
Results of X-ray examination of the rat knee joints. Arrow “a and “c”: sparseness of metaphyseal bone trabecula; arrow “b” and “g”: blurring or premature closure of the epiphyseal plate; Arrow “d”: abnormal sclerosis of the epiphyseal plate; arrow “e”: a developmental deformity of the femoral condyle; arrow “f”: defect and irregularity of the femoral condyle

In group A: the morphology of tibial plateau and femoral condyle were normal, the trabeculae of metaphysis were clear and dense, and the arrangement was regular. Group B: The shape of femoral condyle was irregular in the eighth and twelfth week. The obvious bone trabeculae were sparse in the metaphysis. The arrangement of bone trabeculae was disordered. The number of positive cases in the metaphysis and the bone increased with time (Fig. 1). Group C: the trabeculae of metaphysis were sparse, and the density of metaphysis decreased. Group D: the positive changes in the epiphyseal plate were tough, interrupted, sclerotic and with an irregular edge, and the bone trabeculae of epiphysis and metaphysis were sparse and disordered (Fig. 1). After 12 weeks of feeding, the rate of epiphyseal plate necrosis in group D was higher than that in groups A, B and C (*P* < 0.05 for all, Table 1). The total positive rate in the epiphysis, epiphyseal plate and metaphysis in Group D was 78.95% (Table 2, Table 3).

**Table 1:** Comparison of different types of necrosis of epiphyseal plate of knee joint in rats

	Types of necrosis	4th week	8th week	12th week	Total
Control Group (Group A)	Number of rats	*n* = 7	*n* = 8	*n* = 8	*n* = 23
	FN	0	0	1	1
	LN	0	0	0	
	ZN	0	0	0	
	PN	0	0	0	
KBD-affected feed (Group B)	Number of rats	*n* = 6	*n* = 7	*n* = 8	*n* = 21
	FN	1	1	1	11
	LN	1	1	3	
	ZN	0	0	0	
	PN	0	2	1	
commercial feed+ T-2 toxin (Group C)	Number of rats	*n* = 7	*n* = 6	*n* = 8	*n* = 21
	FN	0	1	2	7
	LN	1	1	1	
	ZN	0	0	0	
	PN	0	0	1	
KBD-affected feed+T-2 toxin (Group D)	Number of rats	*n* = 5	*n* = 7	*n* = 7	*n* = 19
	FN	2	1	1	14
	LN	1	2	2	
	ZN	0	1	1	
	PN	0	1	2	

Results of comparison between two groups (*P* < 0.05, χ 2 test with multiple sample rates) were 0.000 (group A vs group B), 0.011 (Group C vs group D), 0.350 (group B vs group C), 0.000 (group A vs group D). *FN* focus necrosis, *LN* lamellar necrosis, *PN* penetration necrosis, *ZN* zonal necrosis

**Table 2:** positive detection rate of epiphyseal and metaphyseal of knee joint in rats (including enlargement of metaphyseal, deformity of epiphyseal, sparseness of metaphyseal bone trabecula, disorder of bone trabecula arrangement, thinning of bone cortex)

	4th week	8th week	12th week	Total
Group A	*n* = 7	*n* = 8	*n* = 8	*n* = 23
	0(0%)	0(0%)	1 (12.50%)	1 (4.35%)
Group B	*n* = 6	*n* = 7	*n* = 8	*n* = 21
	1 (16.67%)	4 (57.14%)	5 (62.50%)	10 (47.62%)
Group C	*n* = 7	*n* = 6	*n* = 8	*n* = 21
	1 (14.29%)	2 (33.33%)	3 (37.50%)	6 (28.57%)
Group D	*n* = 5	*n* = 7	*n* = 7	*n* = 19
	3 (60.00%)	4 (57.14%)	7 (100%)	14 (73.68%)

**Table 3:** Positive detection rate of epiphyseal plate of knee joint in rats (including epiphyseal plate stenosis, perforation, closure and coarseness)

	4th week	8th week	12th week	Total
Group A	*n* = 7	*n* = 8	*n* = 8	*n* = 23
	0(0%)	0(0%)	2 (25.00%)	2 (8.70%)
Group B	*n* = 6	*n* = 7	*n* = 8	*n* = 21
	2 (33.33%)	3 (42.86%)	5 (62.50%)	10 (47.62%)
Group C	*n* = 7	*n* = 6	*n* = 8	*n* = 21
	1 (14.29%)	2 (33.33%)	4 (50.00%)	7 (33.337%)
Group D	*n* = 5	*n* = 7	*n* = 7	*n* = 19
	3 (60.00%)	5 (71.43%)	7 (100%)	15 (78.95%)

#### MRI results for the rat knee joints

**Fig. 2:**
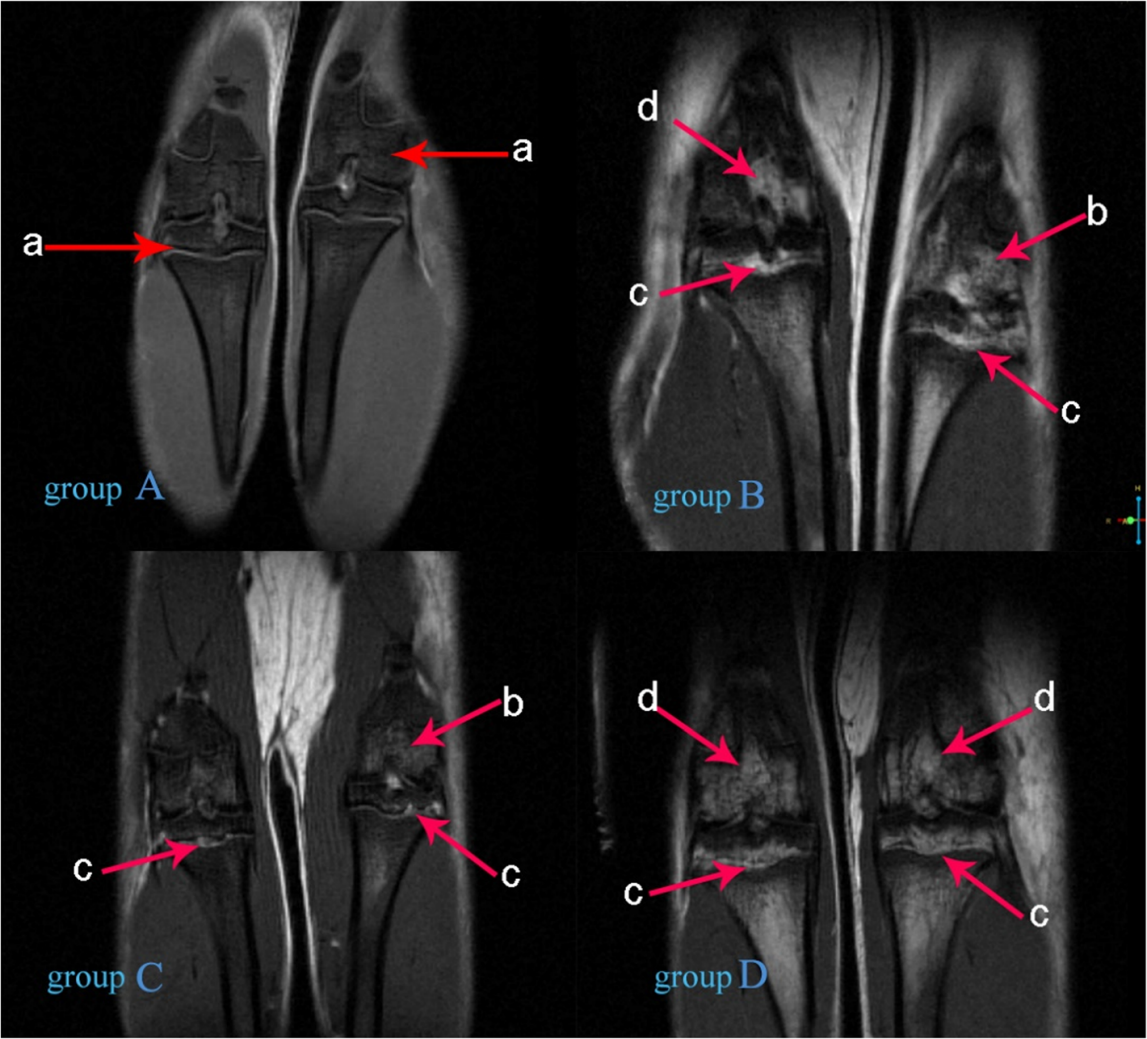
Results of 7.0 T MRI coronal scan of the rat knee joint. Arrow “a”: clear structure and uniform signal in epiphysis, epiphyseal plate and metaphysis; arrow “b”: abnormally high signal in epiphysis; arrow “c”: abnormally high signal in epiphyseal plate; arrow “d” abnormally high signal in metaphysis

In the experimental groups (groups B, C, and D), the primary lesions of KBD involved the epiphyseal plate and epiphyseal end. Compared with the control group, the structure of the epiphyseal plate in the experimental group was not clear on 7.0 T MRI, the signal intensity of the epiphyseal plate was uneven, the continuity of the epiphyseal plate was interrupted and the epiphyseal plate on both sides of femur and tibia showed hyperintense on T1-weighted MR images from point to large area, and the signal mainly extended from epiphyseal plate to the joint surface. The positive ratio of abnormally hyperintense on T1-weighted MR images in group D was higher than that of groups A, B, and C (Fig. 2). At the 4th week, the total positive rates in the epiphysis, epiphyseal plate, and metaphysis in the experimental groups were higher than that detected by X-ray (*P* = 0.001; Table 4). This change in these locations was also confirmed by histological observations. In particular, early closure and fusion of epiphyseal plates were observed on T1WI on the knee joint images of group D rats.

**Table 4:** Comparison of the positive rate of X-ray and MRI in the experimental group at the 4th week

		X-ray	7.0 T MRI	*P* value
Group B (*n* = 6)	Number of positive	2	5	0.135
	Number of negative	4	1	
Group C (*n* = 7)	Number of positive	1	5	0.032
	Number of negative	6	2	
Group D (*n* = 5)	Number of positive	3	5	0.256
	Number of negative	2	0	
Total (*n* = 18)	Number of positive	6	15	0.001
	Number of negative	12	3	

*P = 0.001, the sensitivity of 7.0 T MRI at 4th week was higher than that of X-ray

**Table 5:** Measurement results of the full thickness of epiphyseal plate of tibia in rats (um)

	full thickness of the epiphyseal plate (4th week)	full thickness of the epiphyseal plate (8th week)	full thickness of the epiphyseal plate (12th week)
Control Group (Group A)	539.61 ± 27.59	462.18 ± 21.65	387.55 ± 24.18
KBD-affected feed (Group B)	469.79 ± 31.55	407.78 ± 34.21	328.11 ± 23.62*
commercial feed+ T-2 toxin (Group C)	488.62 ± 30.50	394.99 ± 25.45	338.96 ± 31.44^§#^
KBD-affected feed+T-2 toxin (Group D)	453.92 ± 27.26	346.87 ± 33.80	279.22 ± 17.48^&¥$^

*: *p* = 0.000 vs. groupA; §: *p* = 0.002 vs. groupA; &: *p* = 0.000 vs. groupA; #*p* = 0.395 vs.group B; ¥: *p* = 0.03 vs.group B, $: *p* = 0.000 vs.group C^−^χ ± S

### Histological results (H&E stain)

**Fig. 3:**
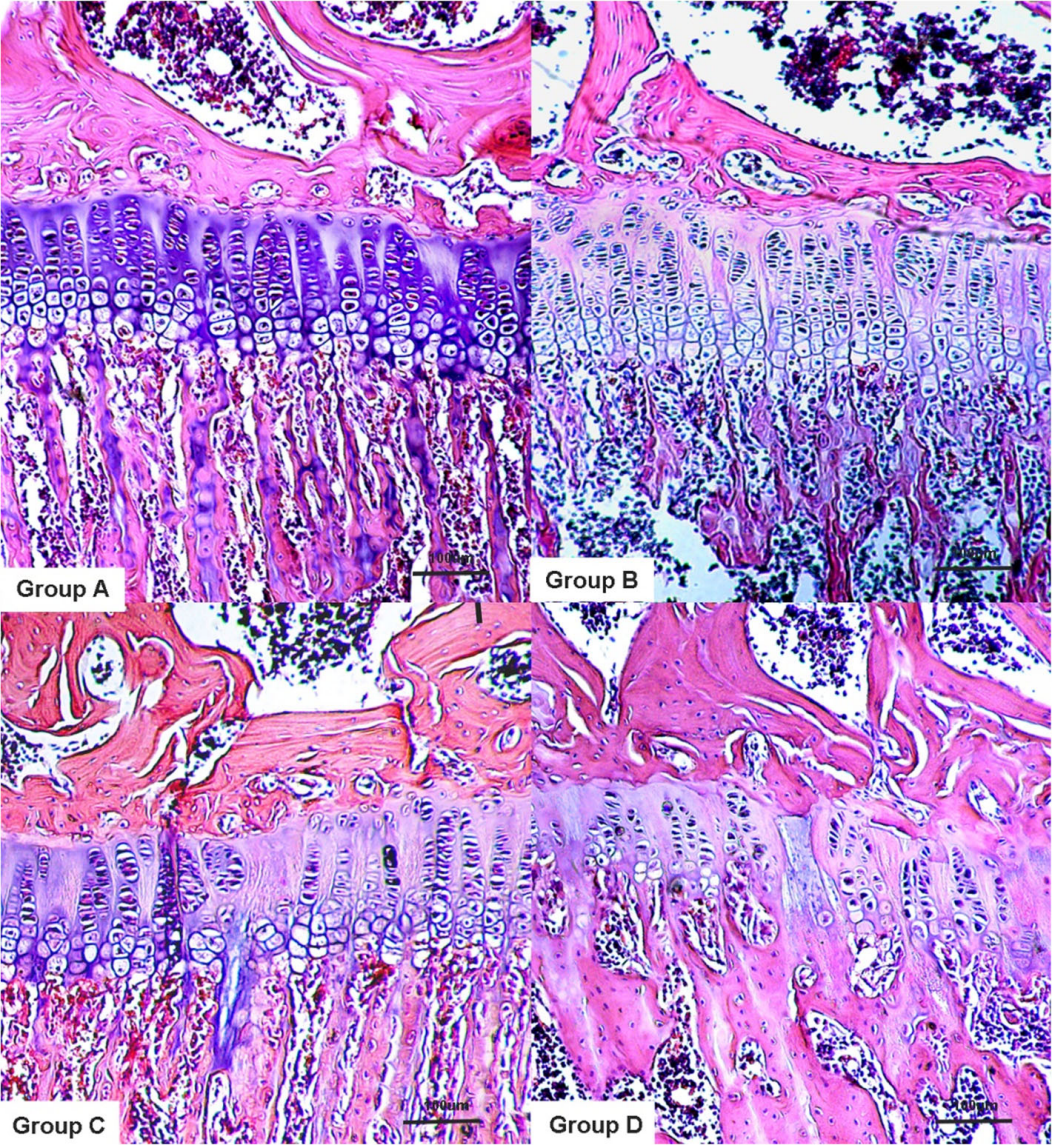
Histological observation of the proximal tibial epiphyseal plate of the rat. Group A: the chondrocytes of the epiphyseal plate are arranged orderly with a clear cytoplasm and nucleus. Groups B and C: focal necrosis of mast cells was found. Group D: the chondrocytes of the epiphyseal plate are disordered with a short cell column, focal necrosis and penetrating necrosis

After 8 weeks of feeding, the tibial epiphyseal plate of the rats in group D (KBD-affected feed + T-2 toxin) showed chondrocyte necrosis with a significant hypertrophic layer and various necrosis types (Fig. 3). In the early stage, chondrocyte focal necrosis was the main necrosis type. As the intervention time increased, the pathological changes in the epiphyseal plate of the rats became further aggravated. The chondrocytes of the epiphyseal plate of the hypertrophic layer necrotized and formed necrotic areas (Fig. 3). The proliferative layer cells adjacent to the hypertrophic layer then degenerated and also became necrotic. The necrotic area expanded and even penetrated the whole epiphyseal plate. The shape of the epiphyseal plate around the necrotic area changed, the chondrocytes of the proliferative layer accumulated, bone maturation was blocked, and the hypertrophic layer atrophied. The thickness of the whole epiphyseal plate was obviously narrowed, and the column of chondrocytes was shorter under the microscope. There was penetration necrosis perpendicular to the epiphyseal plate.

## Discussion

Ultra-high field MRI (7.0 T) may be the most reliable and useful imaging modality for evaluation of the epiphyseal plate, which is otherwise difficult to evaluate because of its anatomical complexity and small size. In this study, typical pathological changes and abnormal MRI imaging characters were found at the epiphyseal plate or epiphyseal end in the rat model of KBD. The epiphyseal plates of rats fed KBD-affected feed combined with T-2 toxin showed hyperintense on T1-weighted MR images [26, 27], and histological observation also showed necrosis of the epiphyseal plate chondrocytes. Compared with the control group, the structure of the epiphyseal plate in the experimental group was rough on 7.0 T MRI, the signal intensity of the epiphyseal plate was uneven, the continuity of the epiphyseal plate was interrupted and the epiphyseal plate on both sides of femur and tibia showed hyperintense on T1-weighted MR images from point to large area, and the signal mainly extended from epiphyseal plate to the joint surface. After the rats had been fed for 4 weeks, X-ray showed positive changes in 6 cases and negative changes in 12 cases, while Ultra-high-field MRI showed positive changes in 15 cases and negative changes in 3 cases; the positive discovery rate was significantly different between the two modalities (*P* = 0.001). Therefore, the research results showed that MRI, especially Ultra-high-field MRI, can show the different anatomical structures (articular cartilage, epiphyseal end, epiphyseal plate and metaphyseal end) of the rat knee joint well and can be used to detect abnormal changes in KBD in the early stage. Especially in the T1 sequence, we found a large number of abnormal hyperintense on T1-weighted MR images in the epiphyseal plate and metaphyseal end of the proximal tibia of the rats in group D. The clarity of the epiphyseal plate was reduced, and the signal of the plate had fused with the surrounding tissue signals. This is strong evidence for the presence of lesions of the epiphyseal plate in KBD. These findings suggest that in the early stage of KBD, we can find abnormal changes in bone and joints by high-field MRI and further judge and differentiate KBD according to the characteristics of the changes. Early detection and diagnosis of KBD is the most critical aspect of further treatment or prevention of disease development.

KBD is caused by various factors, such as diseased food [28, 29], low-nutrient elements, and T2 toxin [30, 31]. T-2 toxin plays an important role in the pathogenesis of KBD [20, 23, 30, 32]. Abnormal changes in the epiphyseal plate were observed in rats fed T-2 toxin and commercial feed, but the pathological changes were not typical. Typical pathological changes were found in the rats treated with KBD-affected feed combined with T-2 toxin, and the necrosis rate and degree were more serious than those in group C (commercial feed and T-2 toxin diet). The thickness of the whole epiphyseal plate was obviously narrowed, (Table 5) and the column of chondrocytes was shorter under the microscope. The main reason for these changes was that the proliferative layer and the hypertrophic layer had become shorter simultaneously. However, compared with the proliferative layer, the hypertrophic layer had shortened further. This is similar to the research results of Kang et al. [23] in 2013. The results of our study thus suggest that T-2 toxin has a particular role in the pathogenesis of KBD. T-2 toxin alone cannot replicate a typical animal model of KBD, and other factors in the diet must also play a role in the disease.

Low nutritional factors maybe another factor which play an important role in the pathogenesis of KBD. There were different degrees of epiphyseal plate lesions in group D, and the changes were more serious than those in group C. The KBD-affected feed has low protein, fat, vitamin, and microelement content, which may be important for the pathogenesis of the disease. The pathological changes of the rats in group D were more serious than those in the other groups, which indicated that the low nutrient elements played an important role in the pathogenesis of KBD. At the same time, epidemiological investigation results [33, 34] and several previous studies [20, 35–37] showed that low-nutrient elements were important prerequisites for the pathogenesis of KBD. Therefore, we speculated that the low nutrient elements might form the basis of KBD.

KBD-affected feed, low-nutrient elements, and T-2 toxin [30, 32, 38] are common factors that related to the pathogenesis of KBD. The results showed that only focal necrosis and lamellar necrosis were found in the epiphyseal plate of the rats treated with T-2 toxin combined with commercial feed. Typical pathological and imaging changes were found in the epiphyseal plate of the rats after 8 weeks, which suggested that T-2 toxin plays a part in the pathogenesis of KBD. Following the consumption of low nutrient-element feed (low levels of protein, selenium, iodine, etc.), pathological lesions are the most serious symptom and form under the joint action of various pathogenic factors, which eventually leads to the onset of KBD. Although this rat model can well simulate the pathological characteristics of KBD, it still has some limitations. The genetic and physiological aspects of rats and human are not exactly the same, and the husbandry conditions cannot simulate the climate and environment of KBD epidemic area.

## Conclusion

Typical pathological changes and abnormal MRI imaging characters were found at the epiphyseal plate or epiphyseal end in the rat model of KBD. MRI at 7.0 T is highly sensitive to the early pathological changes of the epiphysis, epiphyseal plate, and metaphyseal end of the knee joint in rats. This imaging modality can be used for research on early joint lesions and for early diagnosis of KBD. The combination of a low-nutrition diet and T-2 toxin exposure may play an important role in the etiology and pathogenesis of KBD.

## Supplementary information

**Additional file 1: Table 6.** Comparison of the nutritional components between commercial feed and KBD-affected-feed. **Table 7** Changes in hair, activity level and body weight in each group.

## Data Availability

The datasets used and/or analyzed during the current study are available from the corresponding author on reasonable request.

## Abbreviations

KBD: Kashin-Beck disease
MRI: Magnetic resonance imaging
ANOVA: One-way Analysis of Variance
HE: Hematoxylin-eosin
FN: Focus necrosis
LN: Lamellar necrosis
PN: Penetration necrosis
ZN: Zonal necrosis
T1WI: T1 weighted image
T2W1: T2 weighted image
TR: Repetition time
TE: Echo time
FOV: Field of view
