# Supplementary material for: Magnetic resonance imaging at 7.0 T for evaluation of early lesions of epiphyseal plate and epiphyseal end in a rat model of Kashin-Beck disease
Source: BMC Musculoskelet Disord. 2020 Aug 12;21:540. doi: 10.1186/s12891-020-03559-w (PMC7424673; doi:10.1186/s12891-020-03559-w)
Supplement: Supplementary file 1 — Additional file 1: Table 6. Comparison of the nutritional components between commercial feed and KBD-affected-feed. Table 7 Changes in hair, activity level and body weight in each group. [file 12891_2020_3559_MOESM1_ESM.docx]

**Table 6** Comparison of the nutritional components between commercial feed and KBD-affected-feed.

|  | commercial feed | KBD-affected-feed |
| --- | --- | --- |
| Crude protein（%） | ≥18 | 11.4 |
| Fat（%） | ≥4 | 3.0 |
| Zinc（mg/kg） | ≥30 | 22.4 |
| Iodine（mg/kg） | ≥0.5 | 0.215 |
| Fe（mg/g） | ≥0.1 | 0.196 |
| P % | 0.6-1.2 | 0.109 |
| Calcium % | 1.0-1.8 | 0.008 |
| Selenium（mg/kg） | 0.1-0.2 | 0.062 |
| Lysine | ≥0.82 | 0.42 |
| Methionine + cystine | ≥0.53 | 0.30 |
| Arginine | ≥0.99 | 0.56 |
| Histidine | ≥0.40 | 0.24 |
| Tryptophan | ≥0.19 | 0.13 |
| Phenylalanine + tyrosine | ≥1.10 | 0.84 |
| Threonine | ≥0.65 | 0.38 |
| Leucine | ≥1.44 | 0.78 |
| Isoleucine | ≥0.70 | 0.44 |
| Valine | ≥0.84 | 0.58 |
| Vitamin A（IU） | ≥7000 | <7000 |
| Vitamin D（IU） | ≥800 | <500 |
| Vitamin E（IU） | ≥60 | <7.0 |

**Table 7** Changes in hair, activity level and body weight in each group

| Index | Group A  (n=23) | Group B  (n=21) | Group C  (n=21) | Group D  (n=19) |
| --- | --- | --- | --- | --- |
| sparse and easily shed hair (n) |  |  |  |  |
| Week 4 | 0 | 0 | 0 |  |
| Week 8 | 0 | 4 | 2 | 5 |
| Week 12 | 0 | 5 | 4 | 7 |
| Decreased activity (n) |  |  |  |  |
| Week 4 | 0 | 0 | 0 | 1 |
| Week 8 | 0 | 2 | 1 | 3 |
| Week 12 | 0 | 4 | 3 | 6 |
| Weight (g) |  |  |  |  |
| Week 0 | 72.13±3.44 | 70.68±2.54 | 71.11±3.86 | 71.32±2.26 |
| Week 4 | 124.23±14.23 | 65.04±8.89* | 84.87±11.41* | 64.89±7.67* |
| Week 8 | 226.88±32.44 | 113.65±23.55* | 136.11±24.21* | 106.98±21.21* |
| Week 12 | 292.76±53.86 | 179.20±31.87* | 219.21±36.43*^#^ | 169.91±30.97*^#^ |

Weight values given as mean ± SD, * P < 0.05 versus group A. # P < 0.05 versus groups A and B.
